# A Handy Preterm Infant Incubator for Providing Intensive Care: Simulation, 3D Printed Prototype, and Evaluation

**DOI:** 10.1155/2018/8937985

**Published:** 2018-05-10

**Authors:** Amira J. Zaylaa, Mohamad Rashid, Mounir Shaib, Imad El Majzoub

**Affiliations:** ^1^Department of Biomedical Engineering, Lebanese International University, Beirut, Lebanon; ^2^Neuroscience Research Center, Faculty of Medical Sciences, Lebanese University, Beirut, Lebanon; ^3^The University of Texas MD Anderson Cancer Center, Houston, TX, USA

## Abstract

Preterm infants encounter an abrupt delivery before their complete maturity during the third trimester of pregnancy. Polls anticipate an increase in the rates of preterm infants for 2025, especially in middle- and low-income countries. Despite the abundance of intensive care methods for preterm infants, such as, but not limited to, commercial, transport, embrace warmer, radiant warmer, and Kangaroo Mother Care methods, they are either expensive, lack the most essential requirements or specifications, or lack the maternal-preterm bond. This drove us to carry this original research and innovative idea of developing a new 3D printed prototype of a Handy preterm infant incubator. We aim to provide the most indispensable intensive care with the lowest cost, to bestow low-income countries with the Handy incubator's care, preserve the maternal -preterm's bond, and diminish the rate of mortality. Biomedical features, electronics, and biocompatible materials were utilized. The design was simulated, the prototype was 3D printed, and the outcomes were tested and evaluated. Simulation results showed the best fit for the Handy incubator's components. Experimental results showed the 3D-printed prototype and the time elapsed to obtain it. Evaluation results revealed that the overall performance of Kangaroo Mother Care and the embrace warmer was 75 ± 1.4% and 66.7 ± 1.5%, respectively, while the overall performance of our Handy incubator was 91.7 ± 1.6%, thereby our cost-effective Handy incubator surpassed existing intensive care methods. The future step is associating the Handy incubator with more specifications and advancements.

## 1. Introduction

Preterm delivery is the abrupt occurrence of birth at less than 37th week of pregnancy. During the third trimester, that is, 27–40 weeks of pregnancy when the major fetal development stage occurs, the infant undergoes a dramatic transfusion in their respiratory system which enables them to breathe for the first time. After the third trimester, the fetus is usually set to birth [1]. According to the World Health Organization (WHO) epidemiology, in every 10 new born infants, 1 infant is considered a preterm infant [2]. Fifteen million preterm infants were born in 2010. Of all the 15 million, 1 million infant died due to prematurity. The preterm deliveries were then ranked as the first cause of mortality of preterm infants, during the first month of birth and after birth. It is also globally ranked as the second cause of death for children who did not complete their 5 years [2].

Later, a study revealed that preterm birth rates decreased from 2007 to 2014 due to the decreased number of births by teens and young mothers [3]. They also reported a slight increase in the national preterm birth rate between 2014 and 2015 [4]. In almost all countries with reliable data, preterm birth rates are continuously increasing. Blencowe et al.'s systematic analysis showed a continuous increase in the rate of surviving preterm infants in most of the countries. The average annual rate of change from 2005 to 2010 was maintained to 8%, but still equivalent to 92% preterm death.

In high-income countries, almost all of these reported preterm infants survive. In low-income settings, half of the babies born at 32 weeks or less die due to a lack of feasible, cost-effective care, such as lack in warmth, breastfeeding support, and infection control, as well as the existence of breathing difficulties.

Regardless of the reasons of prematurity, many studies have focused on monitoring the maternal and fetal conditions to reduce and predict the symptoms, thus avoiding preterm deliveries [5–12], while others focus on treating the outcome, that is, prematurity, directly reducing the mortality [13–16].

In order to treat the outcome, intensive care methods existed, such as therapeutic methods and devices available in the market and devices that are under research. They vary according to their design, specifications, and performances. They include, but not limited to, commercial incubators, transportable incubator, embrace warmer, radiant warmer, and Kangaroo Mother Care (KMC) methods [17–19]. However, many drawbacks were associated with the existing intensive care techniques.

Despite the presence of intensive care methods, a study predicted the rate of surviving preterm infants for 2025 to be 9% [20]. As the global anticipated rate of preterm infant's mortality for 2025 is 91%, this drove us to tackle this problem and develop a new preterm incubator prototype to promote the intensive care with a low cost. The aim of our study is to develop and 3D print a new handy, portable, and cost-effective liquid crystal display- (LCD-) based incubator for providing intensive care, especially in middle- and low-income countries. The objective is to make the Handy incubator to be feasible and user-friendly and meet the health requirements for preterm infants. The project focuses on the preterm infants abruptly delivered in the third trimester of pregnancy. The major vital signs including the temperature, Heart Rates (HRs), and the level of oxygen were monitored, and advanced biocompatible materials were carefully chosen for treating the preterm infant.

The remainder of this paper is organized as follows. In Section 2, we provide the existing intensive care methods. In Section 3, we introduce the Handy preterm incubator's materials. In Section 4, we set out the Handy preterm incubator's prototype. In Section 5, we present the results. In Section 6, we discuss the results, and in Section 7, we provide a general conclusion and future work.

## 2. Existing Preterm Infant Intensive Care Methods

After searching PubMed, ScienceDirect, and Google scholar, we summarized the literature review results and we divided them into two categories: open care and closed care.

### 2.1. Closed Care Methods

These methods include the infant incubators available in the Neonate Intensive Care Unit (NICU), an intensive care system that supplies the infant with warmth, in a steady stable way, through a heated air circulation over the skin. After several advancements, the infant incubator comprised humidity control, oxygen supply, and other accessories. The infant incubator could be fixed, mobile, or transportable [21]. However, incubators lack the maternal-preterm bond and are expensive, especially in middle- and low-income countries. This triggered other studies to develop portable, cheaper, and feasible systems used at home [22–24].

The fixed infant incubator commonly used in NICU, due to the presence of a variety of accessories, is capable of treating any case. Fixed incubator is seen as a perfect choice since it is connected to wall supplies and provides suitable environment for the infant. Nevertheless, fixed incubators are extremely expensive and have the same concept of producing warmth by pushing heated air through fans. This technique produces noise, which affects negatively the infant [22]. Although such an incubator records HRs, it uses electrodes that have to be connected to the preterm infant all the time, thereby affecting the fragile skin of the infant [23]. Moreover, lack of breast feeding [24] and lack of mobility make it extremely hard for the infant to pass from one department to another, a reason that led to the invention of mobile incubators [25, 26].

A mobile incubator is a modified fixed incubator which has the same function as the fixed incubator. Mobile incubators have additional wheels, could be transported inside the hospital, merely, and require extra tools to supply the system with electricity and oxygen [25]. These incubators have the same drawbacks as fixed incubators. Although mobile incubators are great solutions when the infant needs to be transported inside the hospital, they are impractical when the infant needs to be transported outside the hospital. For this purpose, transport incubators showed up [22–24].

Transport incubators are small-sized portable incubators which can transport the infant using the car or airplane. Despite the fact that transport incubators are the only option for outdoor premature infant transport, transport incubators have several drawbacks, such as the extremely high cost and heaviness, thermostat failure, and electrical shock hazards [27].

### 2.2. Open Care Methods

KMC is a solution for the defects of preterm incubators, which yields to high disease rates and mortality of preterm infants in hospitals. It provides warmth and breastfeeding by infant-maternal skin contact. This bond/contact ensures the stability of the preterm's temperature. Although KMC was capable of reducing the infants' morbidity compared to conventional incubators [28], it is still restricted to different factors. KMC is not capable of monitoring the infant's temperature, HR, oxygen level, and humidity, which subject the infant to a risk of instability and harmfulness. KMC needs skillful human resources like nurses, which add complexity to the intensive care.

Another open intensive care method is the radiant warmer which functions according to the laws of radiant heat. This device provides the preterm with the necessary radiant energy as an alternative process for conventional convection heating [29]. Radiant warmer comprises a bed, an overhead heating unit, and a temperature sensor [29]. Radiant warmers suffer from a dramatic increase in the heat loss due to the evaporation [30, 31].

Embrace warmers made up of three parts, that is, a baby estimated sleeping bag or infant interface, a compartment of phase change material, and a warmer [32], are great solutions for regulating premature infant's body temperature. Meanwhile, embrace warmers do not provide any monitoring for the infant's essential parameters and lack emergency alarms. Also, they require continuous phase change which causes fluctuation in the infant's temperature and omits any therapeutic support.

All the aforementioned problems led us to develop the new Handy preterm infant incubator.

## 3. Materials of the Handy Preterm Infant Incubator

The novel Handy incubator required several materials and tools due to the diverse contributions that were embedded in it.

At the third trimester, the fetus is almost formed and ready for birth [1, 30]. Thereby, the average size, weight, height, head circumferences, and abdominal circumferences of a premature infant [33] were carefully chosen. Notably, during the last three months of pregnancy, the infant brain remains to expand, so the head circumference increases from around 11 inches (28 cm) to 15 inches (38 cm). Simultaneously, the fetus total body length rises roughly from 15 inches (38 cm) to 19 inches (48 cm). The fetus average weight rises from 3 lb (1.4 kg) to 7.5 lb (3.4 kg) [33].

### 3.1. Electric and Electronic Components

The Handy incubator required the ATmega328 microcontroller [34] to launch and store the data.

Arduino Micro was used to assist the microcontroller, as the microcontroller required an overwhelming setup circuits and assembly language. Arduino Micro assists the microcontroller with regulators, with a framework of free libraries and others. The framework provides easier programming and avoids losing time on low-level programming language and registering addresses [35].

The Atmega328 utilized was soldered with a push button to reset, some LEDs to show data transition and reception, and pins labeled with the corresponding pin. Its rear part allows communication with USB and regulator Integrated Chip (IC) to provide stable voltage to ATmega328.

Also, the oximeter MAX30100 was utilized. It is an optical sensor which carries the Maxim's integrated pulse oximeter and HR sensor. Regulator, thermometer, and microBUSinter-integrated communication (I2C) IC were impeded on the rear to provide a 3.3 V supply, measure the temperature, and provide a serial communication.

UltraFire rechargeable batteries (18,650 Li-ion 3.7 V with 9800 mAh capacity) were used [36]. By referring to (1), the energy stored was 36.26 Wh. Thereby, a set of 4 batteries has been used to achieve 9800 mAh, increase the voltage to 15 V, and obtain a stored energy of 147 Wh.

(1) $$\begin{array}{c} E=V\times C. \end{array}$$

### 3.2. Biocompatible Materials and 3D Printer

Three major biocompatible materials were utilized in our Handy incubator: silnylon, mylar sheets, and bamboo fabric. Silnylon was utilized as an outer layer due to its ultra-light weight, windproof, and capability to isolate the system and the infant from the outer environment [37]. The mylar sheet was used due to its high tensile strength, chemical and dimensional stability, transparency, reflectivity, gas and aroma barrier properties, and electrical insulation [38]. The bamboo fabric was utilized due to its antibacterial property, smoothness, breathable property, and great absorbance of water [39]. The ZONESTAR 3D printer was used to establish our Handy incubator due to its several parameters:

- Frame structure materials including printing speed (40–100 mm/s), maximum printable size (220 × 220 × 220 mm), and nozzle size (0.4 mm).
- Printing material supports: poly-lactic acid (PLA) and others, with a diameter including positioning accuracy in *X* and *Y* (0.01 mm) and in *Z* (0.00025 mm).
- Hot bed power: 12 V, 140 W.
- Printing software: Cura, Repetier-Host Kisslicer, etc.; operating system compatible with Windows, Linux, and Mac.
- Melting temperature: 157–170°C; tensile strength: 61–66 MPa; flexural strength: 48–110 MPa.

Moreover, another advantage of the ZONESTAR 3D printer is the fact that it is based on the Fused Deposition Modeling (FDM) printer, which is common and cost-effective, and provides a customized geometry and higher performance [40].

### 3.3. Heat Transfer Components

Two major heat transfer components were embedded in our Handy incubator: the cartridge heater riprap and hot/cold packs. The cartridge heater riprap was the first source of heat energy, which converts electrical energy stored in the batteries into thermal energy, which is in tern stored and transferred to the infant. Cartridge heaters are made from stainless steel and powered with 12 V DC with a power of 40 Watts. The heating probe has cylindrical shape of 6 mm diameter and 20 mm length. This small probe was chosen to ensure that all the thermal energy is transferred to the gel sack. The second component was the hot/cold pack which is a chemical wax that conserves thermal energy and transfers it to the patient via conductance.

## 4. Prototype of Handy Preterm Infant Incubator

The novel steps for obtaining the prototype of the Handy incubator and the testing steps are provided.

### 4.1. Implementation Steps of the Prototype

**Figure 1:**
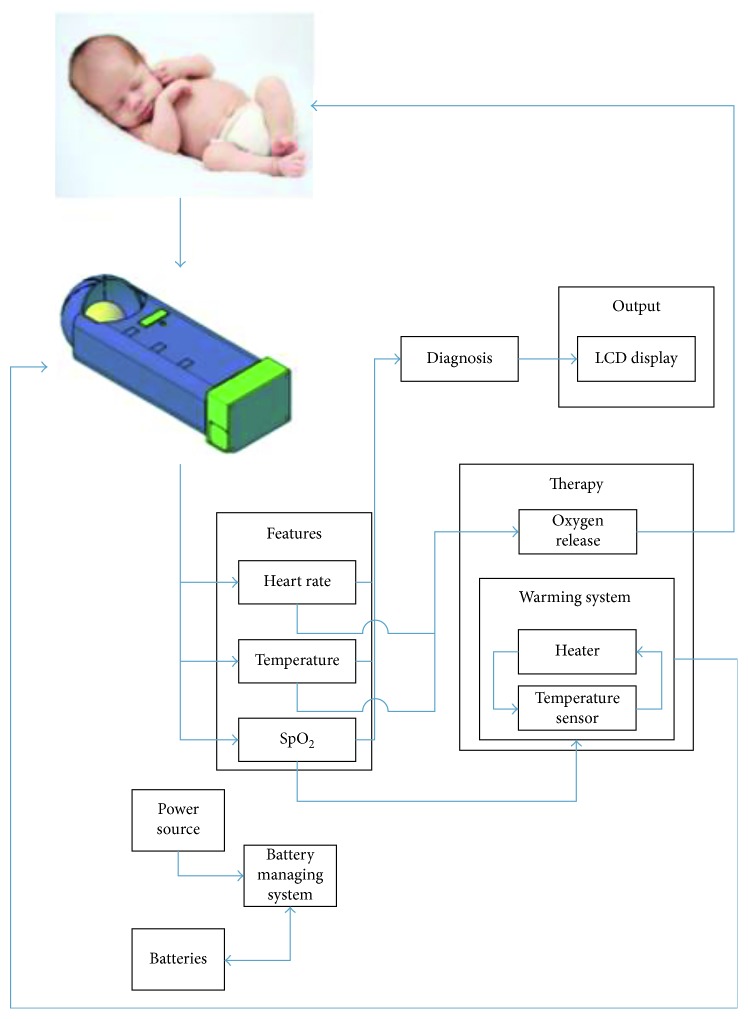
Handy incubator block diagram.

The steps are divided into two major parts: the real and simulated prototype steps and the real prototype testing steps. The block diagram shown in Figure 1 represents our incubator's real prototype steps. After the preterm infant is placed in the novel incubator, three vital signs and features, HR, temperature, and SpO_2_, were continuously monitored (diagnosed) through the microcontroller. Monitored parameters were then displayed on the Handy incubator's LCD. In addition, the power source of the system followed a Battery Management System (BMS).

At any drop out of the normal range of either the oxygen level or the temperature of the preterm, a buzzer is turned on for an emergency interference (therapy), such as releasing oxygen or turning heaters on. This system is supported by a BMS which ensures the mobility of our novel incubator. The simulation steps start by drawing, via AutoCAD, all the required parts present in the block diagram in Figure 1, in accordance with the aforementioned size and the weight of the preterm infant. The block diagram showcases the plan for the desired compact incubator that ensures breastfeeding and can be held by hand (Handy).

Following the simulation steps, the real prototype steps can be reproduced as follows:

- Program the microcontroller to communicate with the sensors and other parts.
- Integrate MAX30100 to ensure the reading from the infant's leg.
- Monitor HR and SpO_2_ biological features noninvasively by MAX30100. MAX30100 measures the absorption of two different wavelengths of light, and it measures the absorbency of pulsed blood by measuring red and infrared waves reflected from hemoglobin (HbO_2_) and deoxy-hemoglobin (Hb). The different intensities are due to their different absorption coefficients.
- Measure the temperature via MAX30100, as it contains a built-in temperature sensor on its chip.
- Process the signal by a low-noise analog signal processing unit.
- Choose the size of the novel prototype to be compatible with the size of a third trimester infant [33
- Prepare the mylar fabric, cut it according to the dimensions, glue the mylar layer on both sides of a cardboard, and mount the silnylon on the top of the mylar.
- Sew the mylar and silnylon to attach them to our Handy incubator.
- Use several sacks of the heating unit; each heating unit comprises a pack of 5 identical sack gels to ensure a uniform heat distribution. Each sack has its own heater and thermistor to regulate the temperature.
- Control the heaters and the temperature reading of each sack by the microcontroller. Such that, when the temperature decreases under a specific threshold, the microcontroller orders the heater to turn ON in the low temperature sack.
- For safety, allow the manual control of the temperature by turning ON/OFF the heaters using push buttons on the LCD, in case of an error in the temperature reading or autocontrol of the temperature.
- Design the novel incubator's circuit (PCB) using EAGLE. EAGLE board design assigns our preferable dimension of the circuit's components in a compact way, traces the connections of these components through copper, and transforms our idealized design into a precise, real dimension and routed PCB.
- Use the ZONESTAR 3D printer to build the Handy incubator's parts. The printer releases beads of heated thermoplastic by the nozzle while it moves, hence building the designed parts in thin layers. Such a printing process is gradually repeated over and over, permitting precise control of the location and the amount of each bead deposit to form each layer. By then, as each layer of the thermoplastic cools, it hardens, gradually building up the Handy incubator's parts as the layers are completely formed. To achieve this point, the following three steps are needed:
  Step 1: Design the novel incubator parts in a 3D modeling software.
    (a) Illustrate the 3D model parts of the novel incubator on AutoCAD, a 3D modeling software.
    (b) Take into consideration every dimension precisely.
  Step 2: Import your 3D model file to a printing software.
    (a) Export the AutoCAD file in a Standard Triangle Language (.STL) file format, so Cura can understand it.
    (b) Cura slices the model, offering us the chance to preview, scale, and adjust its settings.
  Step 3: 3D print the model on your 3D printer.
    (a) Finally, save the slices on a Secure Digital (SD) memory card, which is inserted into the ZONESTAR 3D printer machine, and ready for print.

(xiv) Develop the circuit by adding copper layers, and then sandwich the double layer copper board with the layer image.
(xv) Use an ultraviolet (UV) source to burn the epoxy and clone the circuit on the board.
(xvi) Drill the holes and solder the parts and connectors.
(xvii) Design the user interface to show an LCD and four push buttons, and display the extracted features and the battery state.
(xviii) In case of emergency, associate a beep or alarm when the level of oxygen diminishes.
(xix) Connect a set of 4 rechargeable Li-ion batteries in series, to obtain a 15 V battery of 9600 mAh capacity, and charge the batteries with a 15 V charger when needed.
(xx) Estimate the duration of turning ON the 40 W power heater. Equation (2) shows that the 147 W power can drive the 40 W power heater more than 3 hours 40 minutes, continuously

(2) $$\begin{array}{c} t=\frac{E}{P}. \end{array}$$

(xxi) For electric safety, connect the batteries using a special port, and control the batteries with a BMS to avoid overcharging or overdischarging.

- Finally, save the slices on a Secure Digital (SD) memory card, which is inserted into the ZONESTAR 3D printer machine, and ready for print.

### 4.2. Testing Steps of Handy Preterm Infant Incubator Prototype

As we were keen on monitoring the infants' HRs, we chose MAX30100. By measuring the intensity of Hb and HbO_2_ in blood, we calculated the oxygen saturation (SO_2_). SO_2_, commonly referred to as “sats,” measures the percentage of HbO_2_ binding sites in the bloodstream which is enriched with oxygen [41]. The HR was calculated by calculating the number of beats per minute (bpm). The heart pumps blood via pulsing, this leads to high intensity of cells on the head of each pulse, and then the pulse is detected by detecting a high number of cells. The high intensity in the head of a pulse leads to high reflection, which decreases as the intensity decreases forming pulses.

These pulses can be caught by establishing a threshold, and when the infrared light (reflected signal) exceeds this threshold, the beat is counted. This was tested in the laboratory on a normal man.

The heaters were tested on water, and the temperature sensors were tested on the heated and cooled water. Finally, the battery capacity was tested using a voltmeter.

## 5. Results

### 5.1. Results of Handy Preterm Infant Incubator

After applying all the implementation steps in Section 3, we present the simulated and 3D printed (real) prototype results of our Handy incubator, in addition to the testing and evaluation results.

#### 5.1.1. Simulated Prototype

**Figure 2:**
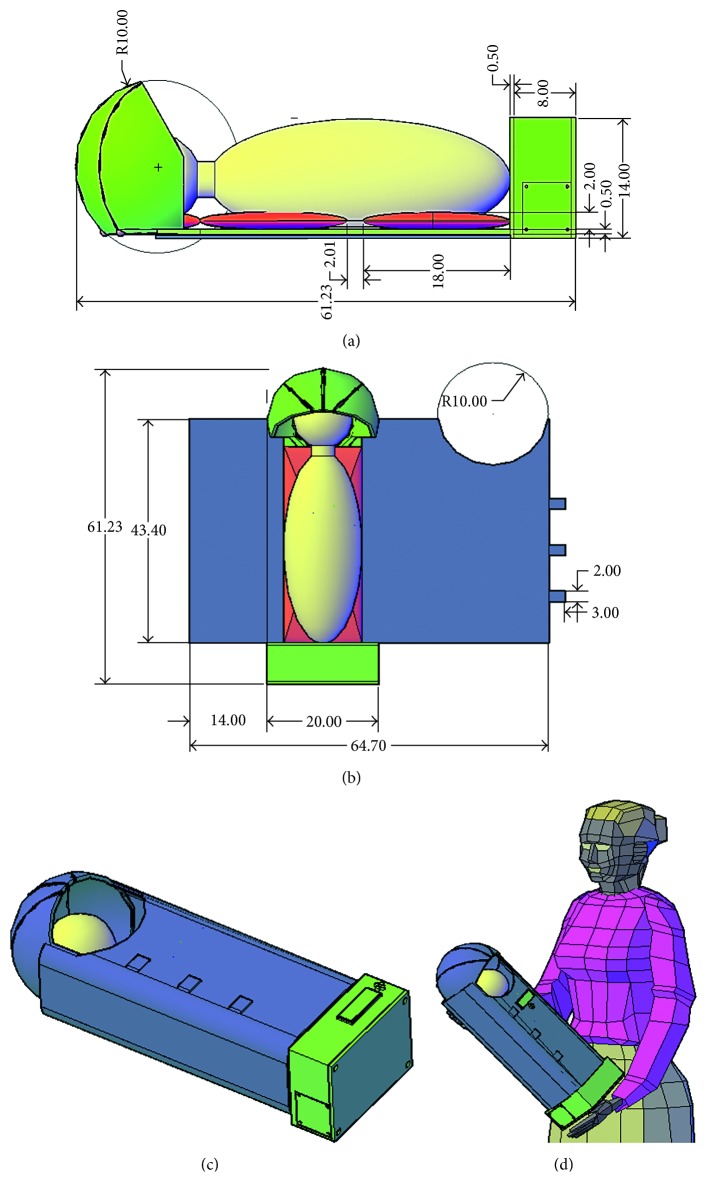
Handy incubator's dimensions drawn using AutoCAD. (a) The real dimensions from a side view. (b) The real dimensions from a top view. (c) The simulated illustration of the closed prototype. (d) The simulated Handy incubator while the mother is holding it by hands.

The simulated prototype of the real dimensions of the Handy incubator is shown in Figure 2(a) from a side view and Figure 2(b) from a top view (in centimeters). A plastic shield, gel pack, and the infant are colored green, red, and yellow, respectively. The total length of the Handy incubator is 61.23 cm, and the length of the box is 8 cm (included within the 61.23 cm). The plastic shield thickness is 0.50 cm, and the mylar and bamboo layers are 0.55 mm each. The thickness of the gel pack is 2 cm. The blue, green, red, and yellow colors in Figure 2(b) represent the outer layer, plastic shield, gel pack, and the infant, respectively. The outer layer surrounds the infant; thereby, it contains a hall of radius 10 cm and three small rectangles. The purpose of the hall was to give the infant the space to inhale oxygen from the environment and ensure breastfeeding. The three small rectangular fabrics were used to hold the two ends of the fabric.

The closed simulation of the Handy incubator is represented in Figure 2(c), where the infant (colored in yellow) is placed inside and surrounded by the outer layer (colored in blue). The green rectangle and four circles on top of the box are the LCD displays and push buttons.  Figure 2(d) shows the overall size of the simulated Handy incubator while the mother is holding it by hands.

**Figure 3:**
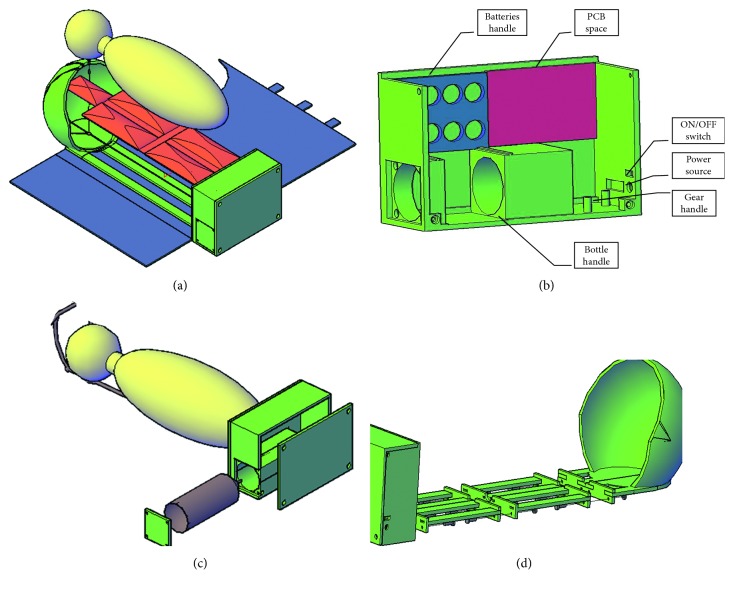
Handy incubator's simulated parts drawn in AutoCAD. (a) The base part. (b) Box label. (c) The warming part/unit. (d) The oxygen source and release parts of the simulated handy incubator.

The novel incubator's simulated base part is shown in Figure 3(a), and the box label is shown in Figure 3(b). The red space represents the position where the PCB is secured. The blue part represents the batteries handle; batteries handle can withstand up to eight batteries. Moreover, the box contains two large holes for oxygen bottle fixation, a hole for the power source deluge, and ON/OFF switch as well as a gear handle that fixes the gear in its place using screws. Figure 3(b) provides the simulation of all the parts required to form our novel incubator. The plastic shield forms the skeleton of our handy incubator (its total length is around 62 cm). The plastic shield was decomposed into four parts connected with screws and nuts.  Figure 3(c) shows the simulation of the warming unit, the red object is the package that represents the gel sacks, and the blue object is the fabric surrounding the preterm. The gel package comprises 5 sacks; each sack is composed of a gel, in addition to a heater and a thermometer to control the generated heat.

The oxygen release part (shown in Figure 3(d)) was simulated to be above the infant's face, by means of a tube-like mechanical valve, a stepper motor with a gear and an oxygen bottle. The oxygen source is colored in brown, and the oxygen bottle was simulated inside the box. Notably, the oxygen transmission tubes are embedded inside the plastic shield to avoid any crash from the external mechanical load.

#### 5.1.2. Preliminary 3D Printed Prototype

**Figure 4:**
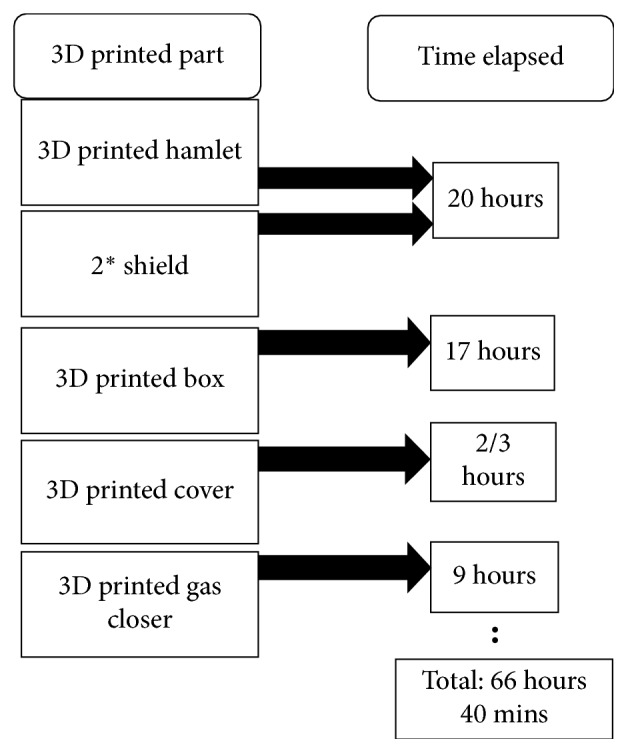
The time elapsed for 3D printing every part of the Handy incubator.

3D printing was the second step towards obtaining the real prototype parts. AutoCAD files were imported to the 3D printer by the means of a memory card to print out the parts. The model and printing duration are reported in Figure 4. The hamlet took ∼20 hours. The box label took ∼17 hours 40 minutes, the cover of the box ∼20 hours, and the two shields 20 hours. The total duration to get all the parts printed was 66 hours 40 minutes.

**Figure 5:**
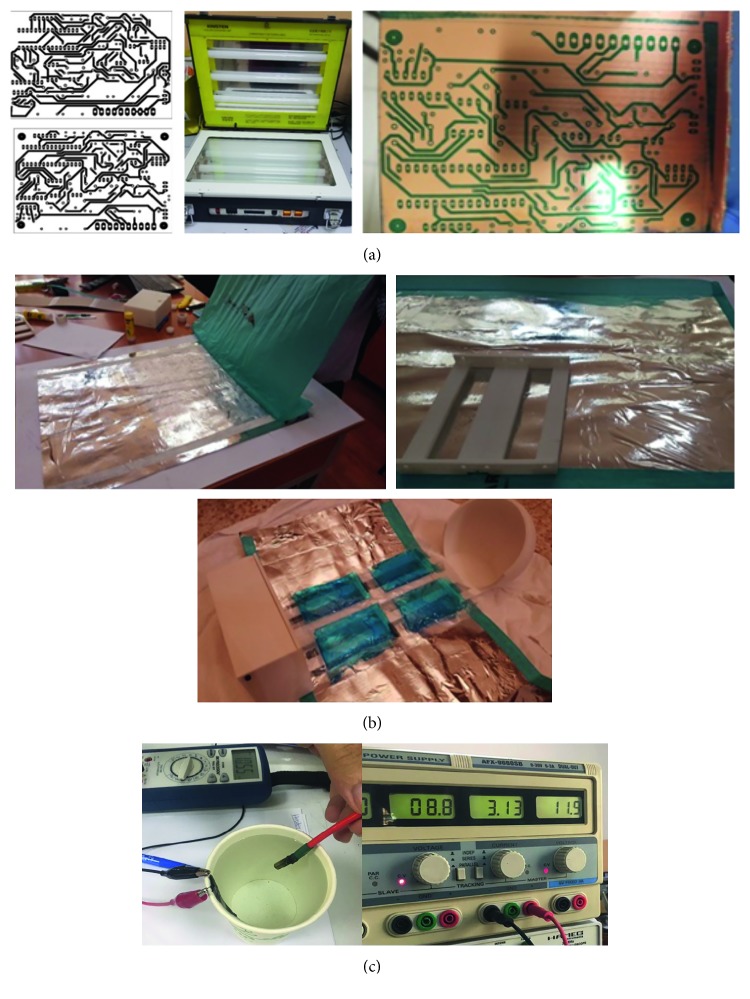
Demonstration of the real prototype's implementation and testing. (a) PCB process. (b) Sewer process. (c) The laboratory setup utilized for testing the warming system component's of the Handy incubator.

The fabric layers sewing, assembly, and circuitry are illustrated in Figures 5(a) and 5(b).

The PCB process is represented in Figure 5(a), from both the bottom and top layers that were printed, UV light source, to the PCB board after washing it with water. The sewing steps are shown in Figure 5(b). It represents gluing mylar with cardboard, the outcome of glued silnylon with mylar and card box, how the obtained fabric is attached to the Handy incubator, and the bamboo fabric which is held on top of the gel packs where the infant lies in the open Handy incubator.  Figure 5(c) represents the laboratory setup utilized for testing the warming system components.

**Figure 6:**
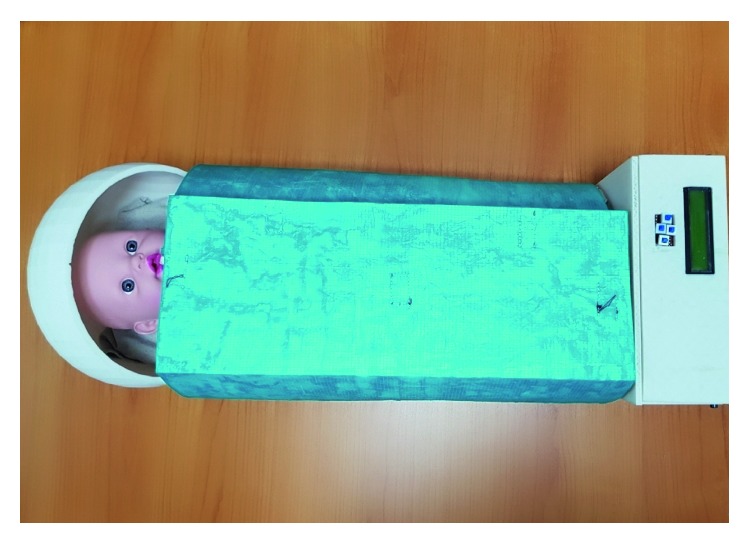
The novel Handy preterm incubator when in a closed mode.

The overall real prototype of the Handy incubator is shown in Figure 6 (closed form). The blue color of fabric is the color of the silnylon that is the outer layer. At the boundaries of the infant surrounding fabric, there are stick tags that provide easy opening and closing of the system. Also the bamboo fabric is attached to the infant surrounding fabric using stick tags; thus, the bamboo fabric can easily be removed, cleaned, and reinstalled.

### 5.2. Testing Results of Handy Preterm Infant Incubator

After presenting both the hardware parts of the handy preterm infant incubator, we present the testing and debugging processes: (i) the electric testing results for the batteries that are intended to supply the system, (ii) the thermal energy released and the warming system, and (iii) the infrared testing. Also, the evaluation and management of the Handy preterm incubator's specifications and cost are provided and compared to the existing intensive care methods.

Electrical testing of the capacity of batteries was obtained by fully charging the battery (until the battery voltage reached 4.2 V), producing a simple circuit that needs specific current (known as the testing current), and measuring the time needed to completely discharge (until the battery voltage reached 2.5 V), which was the capacity.

The test was repeated on UltraFire TR 18650 5 Ah 3.7 V with the testing currents *I* ∈ {0.2,  0.5,  1,  2,  3,  5 A}, and the results obtained were 1.124, 1.123, 1.095, 1.052, 0.955, and 0.626, respectively, and the capacity was not enough. For that, we used two sets of series batteries connected in parallel instead of a single set, to achieve an energy of 23.855 kJ. This energy was capable of heating the system one time and can maintain the warmth for about 16 hours.

The results of both the warming system embedded in the Handy incubator and the thermal energy testing were provided in our previous publication [42].

Pertaining to the insulation, the incubator's fabric and biocompatible materials provided a good insulation.

Infrared testing included MAX30100, and the results were compared to those of oximetry sensors used in mobile phones, a medical equipment specialist for monitoring SpO_2_, and HRs using oximetry sensors. MAX30100 results were reliable and closer to the medical equipment than to the mobile sensor.

### 5.3. Handy Preterm Infant Incubator Evaluation versus Preterm Infant Intensive Care Methods

**Figure 7:**
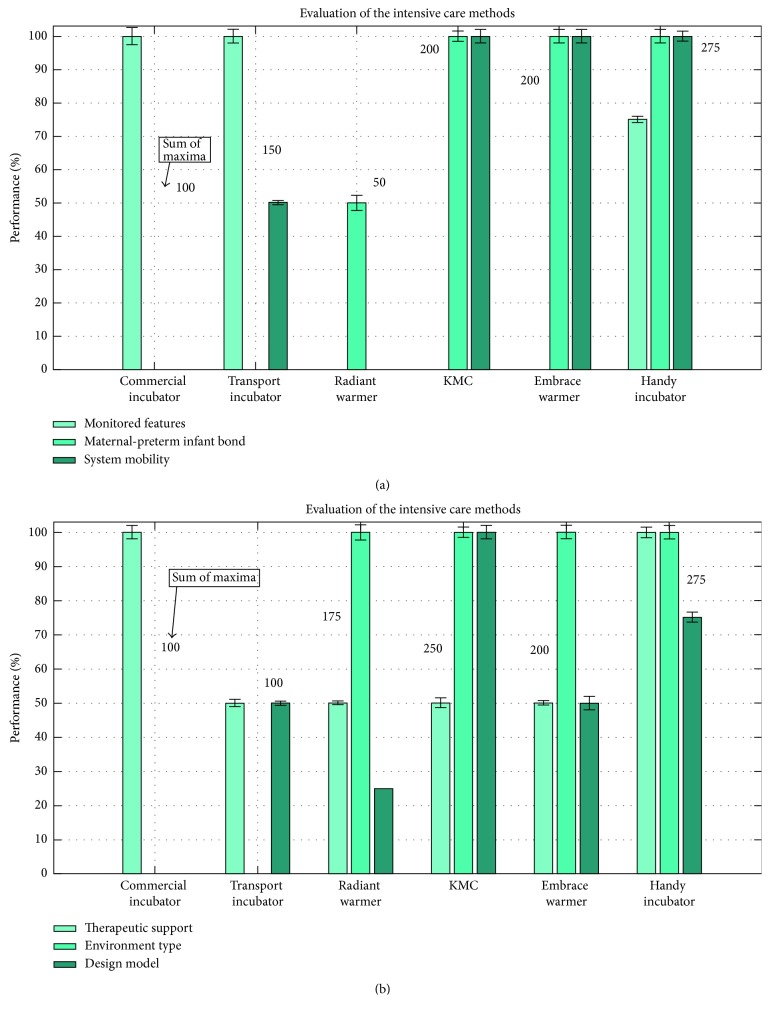
The evaluation of the Handy incubator compared to the intensive care methods: commercial incubator, transport incubator, radiant incubator, Kangaroo mother care (KMC), and embrace warmer. (a) The variation of the monitored features or measurements recorded, the maternal-preterm bond, and the system mobility versus intensive care methods. (b) The variation of the therapeutic support, environment type, and the design model versus intensive care methods.

**Figure 8:**
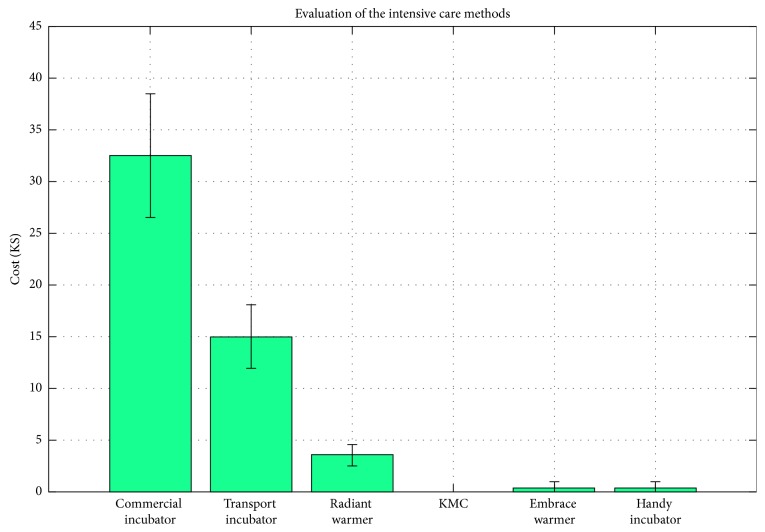
The evaluation of the Handy incubator compared to the intensive care methods: commercial incubator, transport incubator, radiant warmer, KMC and embrace warmer. The bar graph represents the cost (in 1000$).

Evaluation of our Handy incubator included comparing it with peer intensive care methods. Three bar graphs of several crucial factors with the standard deviations imposed on the bar graphs are shown in Figures 7 and 8. These specifications are the price, environment, measurements, mother bond, prototype, mobility, and other factors. Each specification was associated a color in each bar graph, from light green to a dark green color.

Our Handy incubator was compared to the commercial incubator, transport incubator, radiant warmer, KMC, and the embrace warmer, and the results are provided in Figure 7. The variation of the type of monitored features or measurements recorded, for instance, is represented by a bar graph in Figure 7(a) relative to the intensive care methods. The variation of the maternal-preterm infant bond and the variation of the mobility specifications or system mobility versus the intensive care methods are also reported. The variation of the therapeutic support, environment type of the system, and the design model was evaluated and compared with the intensive care methods in Figure 7(b).

The monitored features, evaluated in Figure 7(a), are the vital sign that each method can measure. A maximum value of 100% was associated with the maximum number of features extracted, and a null value of 0% was associated with the absence of any measured feature by the system. The highest number (100%) of extracted features, including SpO_2_, humidity, HR, and temperature, were monitored using both the commercial and transport incubators. Moreover, 75% of extracted features, including SpO_2_, HR, and temperature, were extracted by the Handy incubator, and null otherwise.

The maternal-preterm infant bond, evaluated in Figure 7(a), is the preterm infant contact with the mother. A maximum value of 100% (with a small standard deviation) was associated with the maximum maternal-preterm infant contact ensured by the system. A null value of 0% was associated with the absence of any contact between the infant and mother, that is, when the infant is placed in a fully closed incubator in the NICU. The maternal-preterm infant bond exists fully (100%) in KMC, the embrace warmer, and our Handy incubator. It is totally absent in the commercial and transport incubators.

The system mobility, evaluated also in Figure 7(a), is the capability of mobilizing the intensive care system. A maximum value of 100% was associated with the maximum feasible mobility, and a null value of 0% was associated with a fixed method. The maximum performance of the system mobility was associated with KMC, the embrace warmer, and the Handy incubator.

The therapeutic support, evaluated in Figure 7(b), is the preterm infant contact with the mother. A maximum value of 100% was associated with the maximum therapeutic support and treatment ensured by the system. A null value of 0% was associated with the minimum therapeutic support. The maximum performance (100%) of the therapeutic support was associated with the commercial and Handy incubators.

The environment, evaluated in Figure 7(b), is the nature of the interface of the method with the surrounding. A closed environment is the total isolation of the preterm infant, while the open environment is the insulation permitting the aspiration of the preterm infant from the surrounding ambient air. Notably, the insulation permitting the inhalation was associated the highest performance (100%). The performance of the environment type was maximum in the radiant warmer, KMC, the embrace warmer, and the Handy incubator.

The design model, evaluated also in Figure 7(b), is the capability of mobilizing the intensive care system. The maximum performance (100%) of the design model was associated with KMC, and then 75% was associated with the Handy incubator.

The cost (in 1000$) of the Handy incubator was represented and compared to the cost of the commercial incubator, transport incubator, radiant warmer, and embrace warmer, and the results are shown in Figure 8. The range of the standard deviation is due to the presence of different commercial incubator designs with different specifications. The cost is the average cost of these existing incubators. As shown in Figure 8, the highest incubator's cost is associated with the commercial incubator. Notably, the gross price reported depends on the company and accessories. KMC is costless, and the cost of both the Handy incubator and the embrace warmer is about 300$, while the cost of the commercial incubator is on average 32K$ (it ranges between 1K$ and 55K$).

## 6. Discussion

Various advantages are associated with the existing intensive care methods, whether open care or closed care. Although the commercial infant incubators and fixed, mobile, and transportable incubators conserve a suitable temperature for the infant and monitor the basic parameters, they differ in the weight, size, cost, and compatible accessories [43]. The major advantage of a radiant warmer is the open access care it provides to preterm infants, which supports procedures like endotracheal intubation [44, 45]. This was in accordance with the 100% environment type performance of the radiant warmer observed in our work. However, the overall performance was 37.5 ± 0.9% as reported in Table 1.

KMC is an open care technique, and a recent review reported a 40% reduction in the risk of postdischarge mortality [46]. Other benefits included increased breastfeeding, maternal-infant bonding, and developmental outcomes [47]. This was reflected in the 100% performance of KMC, when studying the presence/absence of the maternal-infant bond. The aforementioned findings and the endorsement of WHO to KMC [48] support the good overall performance of KMC observed in our results (75 ± 1.4%). The absence of the remaining 25% could be due to limiting the lower weight to 800 g as suggested by Lawn et al. [49].

Incubators are rather widely used, most units consist of two operating modes: the air-temperature manual control, and the skin-temperature automatic control [50]. Most units enable the user to measure the relative humidity [51] and provide support of oxygen to the infant when needed [50]. These facts were in agreement with our findings, where the commercial incubator was associated a 100% performance in feature extraction and therapeutic support, with an almost negligible standard deviation.

Regarding the handy incubator prototype information, Fallon involved the use of the cardiopulmonary machine to monitor and display data on an LCD screen [52]. If the infant's HR becomes too slow or too fast, it gives an alarm [52]. Analogous to the work of Fallon, we programmed our Handy incubator to give an alarm when there is a drop in the features extracted.

Recently, scientists in Baby Center published [53] a blood pressure monitor by connecting a miniaturized blood pressure cuff around the infant's leg or arm in order to monitor the blood pressure [53]. Analogous to their work, we used an oximetry and connected the miniaturized blood pressure cuff to the infant's leg.

Our Handy incubator can be easily carried by the mother and affordable in middle-income and low-income countries. Unlike the mOm system provided by James et al. which lacks the maternal-infant bond and breastfeeding [54, 55], in our system, the infant can benefit from the physiological advantage of breastfeeding from one side as provided by KMC [28, 44, 45, 56, 57] and ensures a warm and antibacterial environment from the other side.

Our Handy system also provides the biomedical feature extraction of the preterm HR, temperature, and SpO_2_ level and displays them on an LCD, and this was reflected by the 75 ± 1.5% performance in Figure 7(b). The absence of the approximately remaining 25% is due to the lack of measuring the humidity.

Notably, overuse/underuse of oxygen supply to preterm infants can harm them; thereby, SpO_2_ was monitored in our Handy incubator and was maintained between 90 and 93% to avoid diseases. Pulse oximetry is an advantageous method of oxygenation monitoring, since it is continuous and noninvasive [58].

In case of emergency, we programmed the system to provide temporary oxygen supply. We also ensured to have a cost-effective Handy incubator as compared to other intensive care methods [21–24, 28, 29, 32, 56, 57, 59].

Testing our incubator was necessary to control the quality of the electric, thermal, and graphic design of the incubator.

The Handy incubator provides a good therapeutic treatment such as oxygen supply and warmth. This paves the way for physician to monitor the premature infant state through diagnosing the three vital signs displayed on the LCD and saving it in the memory.

In addition to the nice shape, the system does not produce any noise during turning on or moving, due to absence of fans, and due to the choice of the materials used in fabrication.

The KMC's overall performance (75 ± 1.4%) was better than the embrace warmer (66.7 ± 1.5%) in our explored specifications. However, our Handy incubator surpassed all the intensive care methods, with an overall performance of 91.7 ± 1.6% (Table 1). The Handy incubator is a user-friendly technique. Despite our incubator took time to be 3D printed, its cost was reasonable as compared to expensive commercial incubators. Thereby, the Handy incubators are promising, especially in middle-income and low-income countries.

## 7. Conclusion and Perspectives

Our original research is composed of both hardware and software contributions. The software implementation involved programming the processor platform via Arduino. The hardware execution involved 3D printing the Handy incubator and its circuit and connecting them to Arduino. Our Handy incubator is designed to be portable, not heavy, and cost-effective.

With the progress of our novel 3D printed prototype of the Handy preterm infant incubator, many lives could be saved. Due to the lack of cost-effective intensive care methods for monitoring all vital signs and saving data and the lack of a system that can be held by hands, we took the challenge in designing our handy and cost-effective infant incubator. Our design monitors the vital signals (temperature, HR, and SpO_2_) and displays them. The Handy incubator ensures breastfeeding and is cost-effective. The evaluated percentage of performance shows that it surpasses the existing intensive care methods.

Our system solved many of the challenges, but still there is a margin for more improvement.

Future steps may include the following:

- Collecting more data on the infrared sensor MAX30100 that we assigned in our system to improve the oximetry reading.
- Rendering and updating the code provided by the manufacturer of the sensor that comprises two LEDs and a photoreceptor with a microprocessor, in order to provide specific pulse width and light intensity to meet the medical criteria.
- Using Peltier cell (semiconductor-based electronic component that operates as a small heat pump according to the “Peltier effect”) instead of the heater.
- Modifying the electronic board by adding charge control (maximum power point tracking) to search for maximum power point and by searching for the load resistance resonance with the resistance of supply that has the maximum efficiency of charging.
- Finally, improving the software and providing a web-server for telehealth achievement and research purposes.

## Figures and Tables

**Table 1:** The overall percentage of performance of the commercial incubator, transport incubator, radiant warmer, Kangaroo mother care (KMC), and embrace warmer.

Preterm infant intensive care method	Overall performance (%)
Commercial incubator	33.3 ± 0.8%
Transport incubator	41.7 ± 0.7%
Radiant warmer	37.5 ± 0.9%
Kangaroo Mother Care (KMC)^∗^	75.0 ± 1.4%
Embrace warmer^∗^	66.7 ± 1.5%
Handy incubator^∗^	**91.7 ± 1.6%**

## Data Availability

As we have provided a new invention and an original research applied on this new invention, and our device is also under a prolonged extension for improvement, also we are establishing a collaboration with a Biomedical Engineering company for developing our device, so we left the data confidential until we register this invention in our names.
